# Stable Housekeeping Genes in Bone Marrow, Adipose Tissue, and Amniotic Membrane-Derived Mesenchymal Stromal Cells for Orthopedic Regenerative Medicine Approaches

**DOI:** 10.3390/ijms25031461

**Published:** 2024-01-25

**Authors:** Enrico Ragni, Simona Piccolo, Andrea Papait, Paola De Luca, Michela Taiana, Giulio Grieco, Antonietta Rosa Silini, Ornella Parolini, Laura de Girolamo

**Affiliations:** 1 Laboratorio di Biotecnologie Applicate all’Ortopedia, IRCCS Istituto Ortopedico Galeazzi, Via Cristina Belgioioso 173, 20157 Milano, Italy; simona.piccolo@grupposandonato.it (S.P.); deluca.paola@grupposandonato.it (P.D.L.); michelamaria.taiana@grupposandonato.it (M.T.); giulio.grieco@grupposandonato.it (G.G.); laura.degirolamo@grupposandonato.it (L.d.G.); 2 Dipartimento di Scienze della Vita e Sanità Pubblica, Università Cattolica del Sacro Cuore, 00168 Roma, Italy; andrea.papait@unicatt.it (A.P.); ornella.parolini@unicatt.it (O.P.); 3 Fondazione Policlinico Universitario Agostino Gemelli IRCCS, 00168 Roma, Italy; 4 Centro di Ricerca “E. Menni”, Fondazione Poliambulanza Istituto Ospedaliero, 25124 Brescia, Italy; antonietta.silini@poliambulanza.it

**Keywords:** mesenchymal stromal cells, adipose tissue, bone marrow, amniotic membrane, housekeeping genes, musculoskeletal disorders, orthobiologics

## Abstract

The therapeutic effect of mesenchymal stromal cells (MSCs) has been described for a variety of disorders, including those affecting musculoskeletal tissues. In this context, the literature reports several data about the regenerative effectiveness of MSCs derived from bone marrow, adipose tissue, and an amniotic membrane (BMSCs, ASCs, and hAMSCs, respectively), either when expanded or when acting as clinical-grade biologic pillars of products used at the point of care. To date, there is no evidence about the superiority of one source over the others from a clinical perspective. Therefore, a reliable characterization of the tissue-specific MSC types is mandatory to identify the most effective treatment, especially when tailored to the target disease. Because molecular characterization is a crucial parameter for cell definition, the need for reliable normalizers as housekeeping genes (HKGs) is essential. In this report, the stability levels of five commonly used HKGs (*ACTB*, *EF1A*, *GAPDH*, *RPLP0*, and *TBP*) were sifted into BMSCs, ASCs, and hAMSCs. Adult and fetal/neonatal MSCs showed opposite HKG stability rankings. Moreover, by analyzing MSC types side-by-side, comparison-specific HKGs emerged. The effect of less performant HKG normalization was also demonstrated in genes coding for factors potentially involved in and predicting MSC therapeutic activity for osteoarthritis as a model musculoskeletal disorder, where the choice of the most appropriate normalizer had a higher impact on the donors rather than cell populations when compared side-by-side. In conclusion, this work confirms HKG source-specificity for MSCs and suggests the need for cell-type specific normalizers for cell source or condition-tailored gene expression studies.

## 1. Introduction

Mesenchymal stromal cells (MSCs) gained popularity as a new option for regenerative medicine approaches in a variety of clinical fields [1]. Among the most studied fields of application, musculoskeletal disorders have underprivileged attention due to the dual need for tissue restoration and inflammation management [2]. In this perspective, MSCs’ immunomodulatory and trophic effects observed in both in vitro and in vivo model systems are ascribed to the release of soluble factors and extracellular vesicles (EVs) with therapeutic properties [3]. For these reasons, MSC-based strategies for orthopedic disorders are clinically available in several one-step (MSC-containing products, such as orthobiologics) [4] and actively sifted in pioneeristic two-step (clinical trials with expanded MSCs) [5] approaches. The main challenge is the high heterogeneity of MSCs’ sources. In fact, if MSCs from adult tissues, such as bone marrow or adipose tissue, have been the most studied for a long time, more recently the interest has been also directed toward fetal/neonatal sources (e.g., placenta, amniotic fluid and membrane, umbilical cord, and cord blood). Among them, amniotic-derived products are being extensively investigated for joint and tendon inflammation and healing [6]. Thus, to treat musculoskeletal disorders, both the whole amniotic membrane or expanded MSCs (human amniotic MSCs (hAMSCs)) are possible options that are currently competing with the use of bone marrow-derived (BMAC, bone marrow aspirate concentrate, or BMSC, bone marrow mesenchymal stromal cells) and adipose tissue-derived (SVF, stromal vascular fraction; tissue; mFAT microfragmented adipose tissue, or ASCs, adipose mesenchymal stromal cells) products.

This heterogeneity in MSC-enriched or expanded products leaves the door open for the issue of the most appropriate treatment for the pathology and the patients under the hat of the personalized medicine paradigm [7]. For this reason, a deep characterization and knowledge of the therapeutic products at several levels, from molecular to biochemical and eventually physiological, is mandatory to improve clinical indications [8]. Thus, the reliable evaluation of differences in gene expression of key players, both MSC donor/source-specific and pathology-related, is of utmost importance as the first fingerprinting step. To answer this question, the majority of the MSC-related literature presents reports comparing differential molecular signatures of MSCs from alternative sources, often described in review papers analyzing side-by-side independently generated data from individual MSC types or culturing conditions [9]. In the few cases of direct comparison, especially for qRT-PCR, the pondered choice of the most appropriate normalizers and housekeeping genes (HKGs) has not been addressed. Therefore, the issue of HKG stability in the MSC field remains a matter of debate for the identification of the most stable candidate between donors of the same tissue type or between MSCs from different sources in view of specific therapeutic applications. In fact, the available HKG stability works present in the literature often are related to a single MSC type [10] or compare the most popular MSCs without a focused view of their clinical use [11], with the exception of answering the question of a more defined differentiation potential [12,13] or response to culturing conditions [14,15].

For these reasons, specific HKGs from different MSC sources may be needed, and a thorough investigation of their reliability is mandatory. To answer these questions, in this work, the three MSC types preferentially used in musculoskeletal approaches (ASCs, BMSCs, and hAMSCs) were tested for the stability of five commonly used HKGs (*ACTB*, *EF1A*, *GAPDH*, *RPLP0,* and *TBP*). Expression data were analyzed by four different computational approaches, and a final ranking of stability was obtained for each MSC type or comparison of the different sources. The crucial issue of the most favorable HKG was tested to evaluate the expression of genes coding for factors involved in MSCs’ regenerative potential for osteoarthritis (OA) as a model musculoskeletal disorder. To our knowledge, the results presented are the first related to adipose, bone marrow, and amniotic membrane MSCs for OA-related studies. 

## 2. Results

### 2.1. MSC Characterization

**Figure 1:**
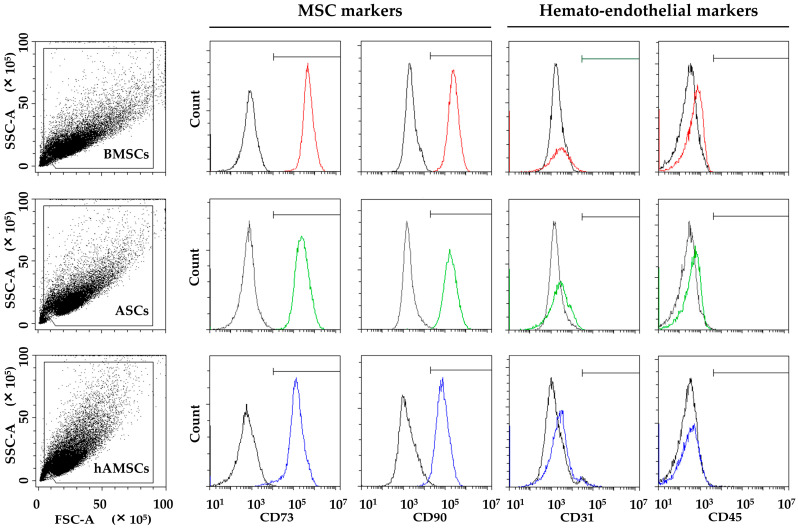
MSC immunophenotype. BMSCs, ASCs, and hAMSCs resulted in positives for MSC markers (CD73 and CD90) and negatives for hematondothelial markers (CD31 and CD45). Plots from a representative donor are shown with colored (red, green and blue) lines for Ab-stained samples.

BMSCs, ASCs, and hAMSCs resulted in positives for the standard MSC markers (CD73 and CD90) and negatives for the haemato-endothelial epitopes (CD45 and CD31) (Figure 1).

### 2.2. Candidate HKGs Expression

**Figure 2:**
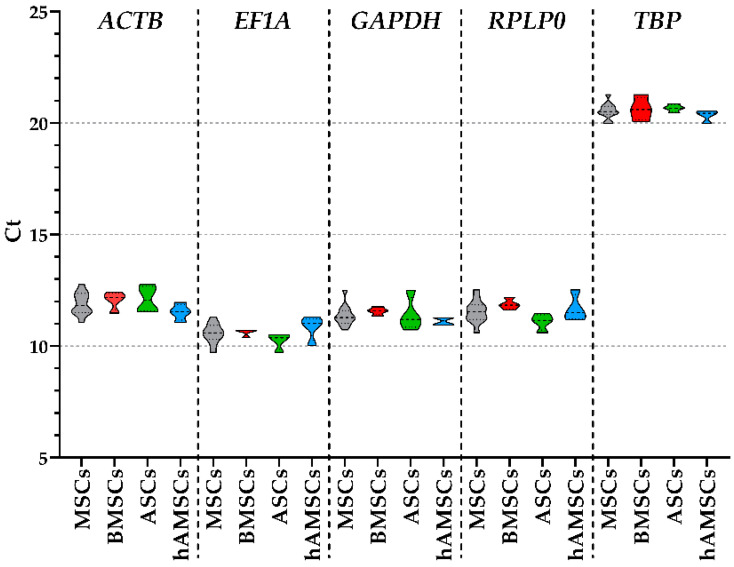
Ct values of the five HKGs across all MSC types tested in the study. Violin plots are shown, with a dashed-line pattern for the median and a dotted-line pattern for quartiles. Gray plots for all MSCs merged together, including blue plots for BMSCs, red plots for ASCs, and green plots for hAMSCs.

Considering all MSC types together, regardless tissue source, *EF1A* resulted the candidate with a lower Ct (10.57 ± 0.12, mean ± SEM with N = 12), followed by *GAPDH* (11.37 ± 0.13), *RPLP0* (11.55 ± 0.14), *ACTB* (11.90 ± 0.14), and finally *TBP* (20.55 ± 0.10) (Table 1 and Figure 2). When the three MSC types were analyzed separately, high stability emerged, with mean Ct values for each gene always ≤1 across all sources (Table 1). These data confirmed the suitability of the selected genes as effective HKGs. Moreover, the five putative HKGs were found located on different chromosomes or, when on the same chromosome, separated by >10 million bases, a value considered as a stringent cut-off for the bonafide absence of co-regulation [15]. In fact, *EF1A/TBP* is on chromosome 6 and *GAPDH/RPLP0* is on the extremities of chromosome 12, with both couples separated by ≥100 million bases, while *ACTB* is on chromosome 7.

**Figure 3:**
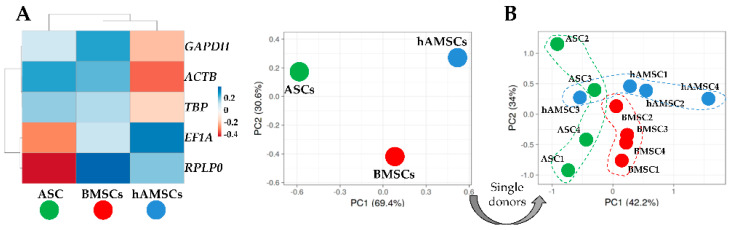
Heat map and principal component analysis (PCA) of HGK expression values across the MSC types. (**A**) Heat map and PCA of samples divided by MSC tissue source. Each value is the result of the four merged donors. In the heat map, negative values mean lower Ct (higher amount), while positive values mean higher Ct (lower amount) with respect to mean values after row centering for each HGK. Both rows and columns were clustered using correlation distance and average linkage. No transformation and no scaling were applied to the dataset. In PCA, the X- and Y-axes show principal component 1 and principal component 2, which explain 69.4% and 30.6% of the total variance, respectively. (**B**) PCA of Ct values of twelve samples (four donors per MSC type). The X- and Y-axes show principal component 1 and principal component 2, which explain 42.2% and 34% of the total variance, respectively.

To obtain further insights into overall HKG expression, a principal component analysis (PCA) and a hierarchical clustering analysis were performed on the mean Ct values of the four donors for the three different MSC types (Figure 3A). PCA showed an equidistant distribution between BMSCs, ASCs, and hAMSCs, with bone marrow and amniotic membrane MSCs under the same node in the heat map. A deeper analysis of the single donors confirmed the presence of distinct clusters depending on the tissue source (Figure 3B), although a less sharp dichotomy emerged, suggesting that a clear definition of MSC type-specific HKGs is mandatory to efficiently allocate reliable molecular profiling.

### 2.3. HKGs Stability

To test HKG stability, four applets were used, and a final ranking was computed (Table 2). First, the three MSCs were analyzed separately. For BMSCs, *EF1A* resulted in the most stable candidate (geomean of the four applets’ rankings: 1.19), while *TBP* was last (5.00). *EF1A* emerged again as the best performer for ASCs (1.32), whereas *GAPDH* did not show high stability, ending at the bottom of the ranking (5.00). Confirming the need to identify source-specific HKGs, *GAPDH*, which underperformed not only in ASCs but also in BMSCs (second-last, 3.36), was the most reliable for hAMSCs (1.32), with *RPLP0* being less reliable (5.00), and the adult-specific EF1A was the second-last in the ranking (3.72).

Due to the diverging stability rankings between adult (BMSCs and ASCs) and fetal membrane-derived (hAMSCs) MSCs, computational analysis was performed on a couple of conditions (Table 2). First, adult MSCs had, as expected, *EF1A* as the most reliable (1.00), and *GAPDH* was at the bottom of the ranking (4.40), which agreed with the single-source analyses. Confirming the different signature between adult and fetal-membrane MSCs, *GAPDH* was a good performer for BMSCs/hAMSCs (first, 1.00) and ASCs/hAMSCs (second, 2.21), together with *TBP* (second for BMSCs/hAMSCs, 2.71; first for ASCs/hAMSCs, 1.00). The adult-HKG *EF1A* was last for BMSCs/hAMSCs (4.40) and second-last for ASCs/hAMSCs (3.46) before *RPLP0* (4.40).

Eventually, all MSCs were analyzed together (Table 2). *TBP* (1.41) and *EF1A* (2.06) were the most stable HKGs, followed by *GAPDH* (2.45), while *ACTB* was the worst (5.00).

### 2.4. Effect of HKG Choice

**Figure 4:**
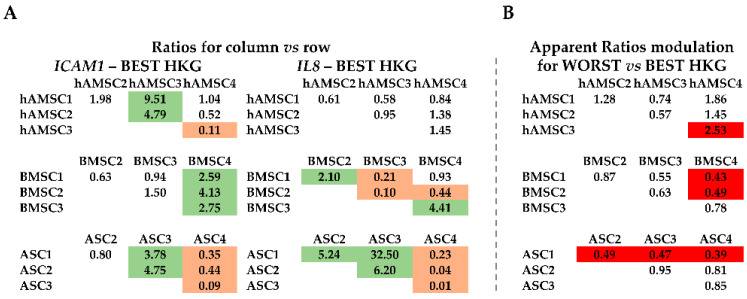
*ICAM1* and *IL8* gene expression comparison within donors of each source-specific MSC type. (**A**) Gene expression ratios between donors of the three MSC types under analysis are expressed as ratios of the donors identified in the column annotation vs. those in the row annotation. Results of the best HKG for each condition are shown. Light green background for ratios >2, orange for ratios <0.5. (**B**) Apparent ratio modulation was obtained by comparing the outcomes calculated with the worst HKG and those with the best normalizer (panel **A**) for each condition. Red background for apparent ratios of either <0.5 or >2.

**Figure 5:**
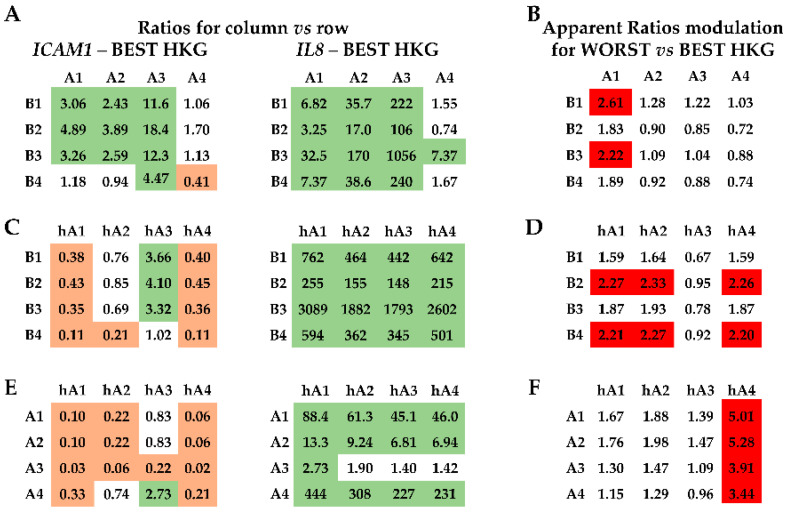
*ICAM1* and *IL8* gene expression comparison between donors of the source-specific MSC types. (**A**,**C**,**E**) Gene expression ratios between donors of the three MSC types under analysis were compared side-by-side and expressed as ratios of the donors identified in the column annotation vs. those in the row annotation. Results of the best HKG for each comparison are shown. Light green background for ratios >2, orange for ratios <0.5. A stands for ASCs, B for BMSCs, and hA for hAMSCs. (**B**,**D**,**F**) Apparent ratio modulation was obtained by comparing the outcomes calculated with the worst HKG and those with the best normalizer (panel **A**) for each comparison. Red background for apparent ratios of either <0.5 or >2. A stands for ASCs, B for BMSCs, and hA for hAMSCs.

**Figure 6:**
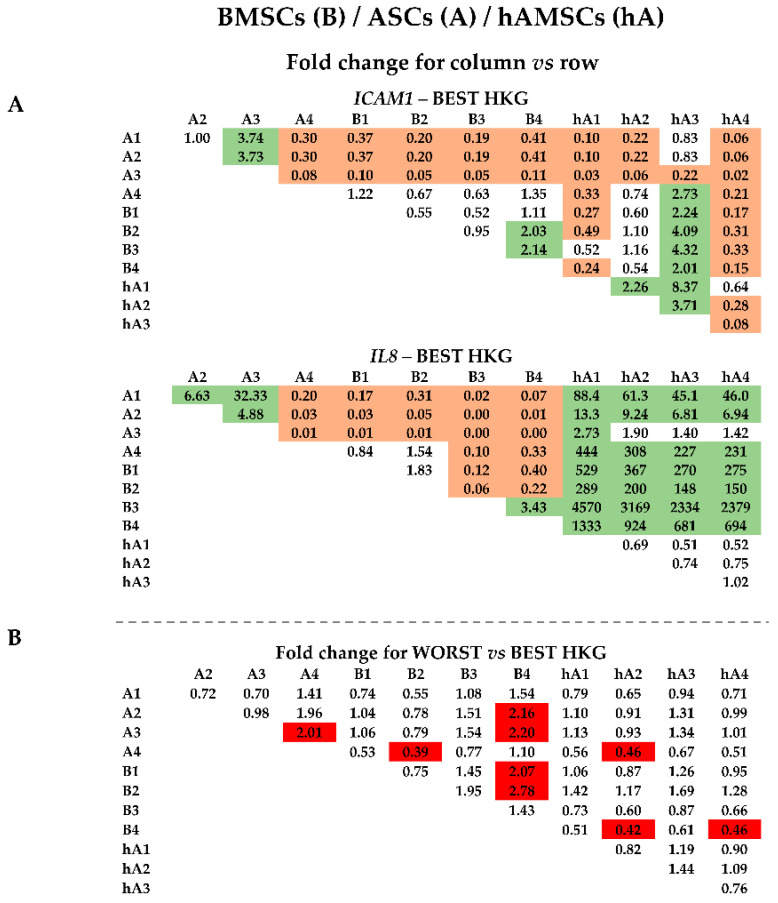
*ICAM1* and *IL8* gene expression comparison between all donors and source-specific MSC types. (**A**) Gene expression ratios between donors of the three MSC types under analysis were compared together and expressed as ratios of the donors identified in the column annotation vs. those in the row annotation. Results of the best HKG for each gene are shown. Light green background for ratios >2, orange for ratios <0.5. (**B**) Apparent ratio modulation was obtained by comparing the outcomes calculated with the worst HKG and those with the best normalizer (panel **A**) for each gene. Red background for apparent ratios of either <0.5 or >2.

To weigh the effect of HKG choice on gene expression, two candidates involved in MSC immunomodulation and paracrine potential were tested: *ICAM1* (Intercellular adhesion molecule-1) and *IL8* (Interleukin-8). First, mRNA amount comparison within each MSC type was performed using the best or worst HKG (Figure 4A,B). A difference between donors for each cell source for both genes (Figure 4A) emerged, confirming the need for reliable HKGs. In fact, when using the less stable normalizer, apparent > 2-fold differences in the mRNA ratios were detected for several comparisons (Figure 4B, red background). As an example, with the worst HKG, the hAMSC4/3 ratio had an erroneous increase, and BMSC4/1 and BMSC4/2 ratios decreased, as well as all comparisons of ASCs related to ASC1. Second, donors of the different MSC types were analyzed side-by-side (Figure 5). *IL8* always resulted in more (>2-fold, with values sometimes close to a thousand-fold upregulation) values expressed in hAMSCs donors, with the exception of ASC donor 3, which had comparable levels with amniotic MSCs (Figure 5A,C,E). Moreover, ASCs, excluding donor 4, had more *IL8* than BMSCs. For *ICAM1*, the situation was less clear, with a general higher (>2-fold) amount for ASC 1-3 vs. BMSC 1-3 and a lower (<0.5-fold) expression for hAMSCs 1 and 4 vs. all BMSCs, a result that was confirmed, including hAMSC 2, in comparison with all ASCs. Confirming higher donor-dependent outcomes, hAMSC 3 had an opposite trend with higher levels than BMSC 1-3. Of note, the use of less stable HKGs resulted again in incorrect ratio modulation, with an apparent reduction for BMSCs 2 and 4 with respect to hAMSCs 1-2-4 and a false increase in hAMSC4 compared with all ASCs and for ASC 1 when compared side-by-side with BMSCs 1 and 3 (Figure 5B–E, red background). Third, all samples were analyzed together (Figure 6). The analysis confirmed the higher *ICAM1* levels in ASCs (Figure 6A), with the exception of ASC 4, and the higher amount of *IL8* in hAMSCs (Figure 6A), with the exception of ASC 3, where high mRNA expression was corroborated. Similar to before, the wrong HKG did not allow for a correct comparison in very many cases (Figure 6B, red background).

**Figure 7:**
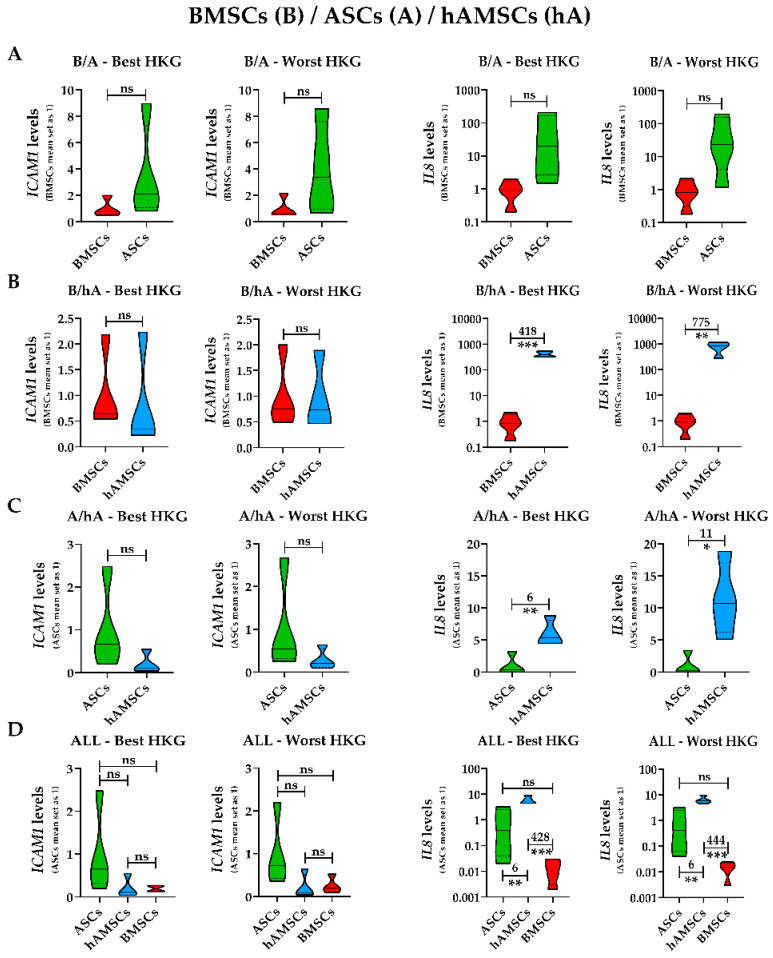
*ICAM1* and *IL8* gene expression comparison between source-specific MSC types with best and worst HKGs. (**A**) BMSCs (B) and ASCs (A). (**B**) BMSCs (B) and hAMSCs (hA). (**C**) ASCs (A) and hAMSCs (hA). (**D**) All MSC types under study. Violin plots of the four donors for each MSC type are shown. ns is not significant, * is a *p*-value < 0.05, ** is a *p*-value < 0.01, and *** is a *p*-value < 0.001. Numbers on the top of the comparison lines indicate the fold change among gene expression means when the statistical significance requirement is met.

Eventually, we investigated the effect of HKG choice under an MSC-type approach, rather than directly comparing the single donors (Figure 7). For gene expression analysis at the cell population level, the HKG effect was greatly reduced since both best and worst normalizers ended up with similar results. No significant (*p*-value < 0.05 with fold change >2) differences were found for *ICAM1* due to donor-dependent fluctuations in gene expression within each MSC type. On the contrary, for *IL8*, if ASCs and BMSCs did not end up with statistic differences (Figure 7A), higher levels were confirmed for hAMSCs when analyzed against either a single MSC type (Figure 7B,C) or all (Figure 7D) MSC types. The only variation observed with the worst HKG was an apparent double *IL8* amount for hAMSCs in the side-by-side comparisons, alongside a weaker statistical significance. Therefore, HKG choice appears more crucial for single donors than a specific cell type.

## 3. Discussion

The present work showed that adult (bone marrow and adipose) and fetal/neonatal (amniotic membrane) MSCs have opposite stable HKGs. Moreover, when MSCs are analyzed side-by-side, a comparison-specific HKG must be selected. These data emphasize the need for source- and setting-tailored normalizers for MSC studies.

For the treatment of musculoskeletal disorders, orthobiologics are a valuable option [16] since they can be obtained in an office setting and are highly enriched in biologic substances and bio-active cellular components, including MSCs. Orthobiologics have shown promising outcomes in managing tissue damage and inflammation, including the amelioration of bone marrow lesions [17,18,19]. These lesions, which are often challenging to treat, can be visualized with advanced imaging techniques, such as spectral CT or MRI [20]. The most popular orthobiologics are based on bone marrow aspirate, adipose tissue, and amniotic membrane [21], and for these reasons, the knowledge of BMSCs, ASCs, and hAMSCs was used to explain the main tissue regenerative and anti-inflammatory properties [22] of these one-step (minimal manipulation at the bedside) products. As a natural step, the idea of using expanded MSCs has been actively studied in several clinical trials, with ASCs [23], BMSCs [24], and hAMSCs [25] among the most promising cell types.

To date, the selection of the most effective treatment based on either minimally manipulated products or expanded MSCs remains an unanswered question. The majority of research articles analyzed the outcomes of no more than a couple of different orthobiologics or expanded cells side-by-side, while available reviews and meta-analyses compared more treatments, albeit from unrelated publications. Although giving valuable hints, the bias of homogeneity in the results is still debatable, especially for direct comparison of molecular data when normalizers are used without verification of their suitability for the specific analysis. This can lead to misleading statements, as demonstrated by our work, which clearly showed how HKGs’ reliability is dependent on the MSC type and source. Adult MSCs (BMSCs and ASCs) had *EF1A* as the best performer, while the widely used *GAPDH* was less performant. Our results are difficult to directly compare with the existing literature since for many reports, HKG candidates were either partially different, or their stability was not assessed for each cell type during expansion separately but merging all datasets [26], or across several conditions such as differentiation [12,27] or expansion in 2D vs 3D scaffolds [26]. Nevertheless, the presented data for BMSCs confirmed and extended what was observed for the same cell type cultured in 2D, where *RPLP0*, *GAPDH,* and *TBP* were found in the identical stability order [26]. Moreover, in another report for BMSCs under expansion, *EF1A* was suggested as the best HKG, and *GAPDH* was very unstable [28]. An interesting result of our analysis was the inverted stability order observed for hAMSCs, where *GAPDH* was the most stable and *EF1A* lost its reliability. This confirms the differences that emerged at the whole transcriptomic level between adult and fetal/neonatal MSCs [29]. For this reason, the choice of the most appropriate HKG has to be carefully evaluated for comparative studies, and a “middle way” approach is not always the winning strategy. In fact, whether for the ASCs/BMSCs couple *EF1A* ranked again first with *GAPDH* last as for the single cell types, for BMSCs/hAMSCs and ASCs/hAMSCs the couple *GAPDH*/*TBP* emerged as the best normalizer while *EF1A* performed again poorly even if a position in between of the ranking for both *GAPDH* and *EF1A* could be postulated based on single cell type analyses. Eventually, when all MSCs were merged, *TBP* was the most stable candidate, with adult *EF1A* and fetal/neonatal *GAPDH* in the second and third positions, respectively.

The effect of poor HKG selection was tested within donors and between sources for each MSC type on the expression amount of *ICAM1* and *IL8* by coding for players involved in MSC activity as therapeutics for osteoarthritis (OA). ICAM1 is a highly glycosylated protein that belongs to the immunoglobulin superfamily of cell adhesion molecules [30]. For MSCs, it was shown that cells with high levels of *ICAM1* have a more pronounced immunosuppressive effect on dendritic and T cells [31], which are both involved with OA pathology [32]. From our data, it clearly emerged that *ICAM1* mRNA levels are donor-dependent within each MSC type, whereas between sources, no statistical differences emerged. The donor difference is a crucial issue since in view of using GMP-compliant MSCs for therapy, the selection of so-called “superdonors” for personalized medicine approaches is a matter of investigation. As an example, a similar result for expanded MSCs in orthopedic research was obtained by studying the donor-dependent chondrogenesis potential in 3D hydrogels for engineered cartilage constructs [33]. Also, for MSC-enriched products, such as orthobiologics, the level of specific molecules in the stromal components might explain or even predict the high or low therapeutic outcomes. A similar donor-related gene expression fingerprint also appeared for *IL8*, a pro-inflammatory chemokine [34] with multiple roles in OA development [35]. In addition, *IL8* amount was also source-specific, with hAMSCs having the highest expression, confirming the literature data [36]. It should be noted that the effect of HKG choice was more impactful for comparing single donors rather than source-specific MSC types. This is in agreement with the proposed role of the HKGs used in this study, which are often used as “general” normalizers but are rarely investigated at the single-donor level. Thus, the similar behavior when comparing cell types confirms their fame as reference genes for cell population molecular studies; although, a thorough investigation must be performed for optimal results when donors are compared.

As a general comment on the results described herein, our data contribute as a brick in the façade of defining the optimal treatments, whether MSC-enriched or MSC-expanded, for musculoskeletal conditions, with a specific focus on OA. In fact, dissecting the heterogeneity in methodologies for characterizing MSC features and potential, at least partially addressed in this work through the identification of the best HKGs for molecular fingerprinting, is crucial. This heterogeneity is further compounded by variations in study design and patient population [21]. The limited number of patients and the simultaneous comparison of only a few orthobiologics or MSCs at a time may compromise the reliability of therapeutic outcomes and impede the identification of the most effective treatment. Despite these challenges, our study aims to extract valuable molecular data from smaller subsets, leveraging robust normalizers to mitigate patient and treatment heterogeneity. It is important to note that while our data help in selecting the most appropriate treatment for a specific patient population, they cannot independently predict the efficacy of a specific orthobiologic/MSCs due to differences in composition, dose, and administration route. As a matter of fact, it is known how different orthobiologics have divergent amounts of MSCs, depending on the tissue source, with amniotic membranes and adipose tissues being more enriched than bone marrow (millions per gram vs. thousands per ml) [37,38]. Moreover, our data provide valuable insights into MSCs and MSCs-enriched products at the point of administration. Nevertheless, to achieve a thorough comprehension of long-term outcomes and safety in these innovative therapies, extended follow-ups over several years are crucial [39]. It is noteworthy that such prolonged follow-up periods are frequently overlooked in clinical studies, but they are imperative for a comprehensive evaluation of the sustained effectiveness and safety profiles of these treatments. This lack of in-depth characterization of cell products and their outcome/safety profile is, therefore, a major drawback for future use of orthobiologics and MSC-based products. It is essential to note that the data presented were obtained using standard expansion protocols for cells. The next generation of media (platelet lysate [40] or serum/xeno-free supplements [41]), alternative culture surfaces (3D vs. 2D or bioreactors [42]), and emerging bionanomaterials [43] are looming on the horizon. Notably, these advancements have the potential to introduce variations in the quality and potency of cells used across different studies. For instance, next-generation media and bionanomaterials can control the release of growth factors and interact with cells at the molecular level to enhance adhesion, migration, and differentiation, ultimately improving the integration of MSCs into host tissues. This evolving landscape underscores the need for future analyses, akin to the one presented here, to reliably characterize new-generation MSC-based treatments, at least on a molecular level. In particular, a pathology-focused analysis, beyond the musculoskeletal disorders discussed, holds promise in refining the effectiveness of MSCs based on the specific pathophysiology of the targeted disorder. As the field advances with innovative methodologies and materials, comprehensive studies will be essential to ensure a thorough understanding of the quality and potency of these evolving cell-based therapies.

We are aware of the limitations of this study. We focused on the most studied MSC types for musculoskeletal regenerative medicine approaches for both orthobiologics and GMP-compliant cells. In recent years, other MSC types, such as iPSC-derived [44], were proposed, and the analysis presented will need a revision after these new sources are extensively studied. Also, the choice of HKGs analyzed in this study reflects a subset commonly described in the literature. We deliberately selected the most widely used ones, including the “gold standard” *ACTB* and *GAPDH*, to facilitate the translation of our results across a majority of laboratories. The demonstrated reliability of these genes as “general” HKGs for population studies validates the appropriateness of our selection. Finally, a limited number of donors was tested. Future studies will be needed to strengthen the reported results, and analyzing samples from clinical studies and those obtained with alternative protocols or devices will be essential. This expanded approach will allow for the integration of molecular data with functional correlations, the consideration of donor-specific variations, and the assessment of clinical relevance. Ultimately, these future studies will contribute to a more comprehensive understanding of MSC behavior and therapeutic potential.

## 4. Materials and Methods

### 4.1. Ethics

This study was conducted in accordance with the Declaration of Helsinki and its later amendments or comparable ethical standards. Institutional Review Board approval was obtained (San Raffaele Hospital Ethics Committee approval on 16 December 2020, registered under number 214/int/2020), and informed consent was given to patients involved in this study.

Human term placentae, utilized for isolating hAMSC, were sourced from healthy women following vaginal delivery or cesarean section at term after obtaining informed written consent, in accordance with the guidelines established by the local ethical committee “Comitato Etico Provinciale di Brescia”, Italy (number NP 2243, 19 January 2016).

### 4.2. Adipose (ASCs), Bone Marrow (BMSCs), and Human Amniotic Membrane (hAMSCs) MSC Isolation and Culture

The bone marrow aspirate of four donors (two males and two females, mean age 45 yo ± 3) was harvested from the anterior-superior iliac crest. A complete blood cell count was performed to assess the number of nucleated cells by an automatic cell counter (NucleoCounter NC-3000, ChemoMetec, Allerod, Denmark). Samples were initially plated at the density of 50,000 cells/cm^2^ in DMEM/F12 supplemented with 10% FBS and 1% PSG. BMSCs able to adhere to plastic were cultivated at 37 °C, 5% CO_2_, and 95% humidity.

Waste adipose tissue of four female donors (mean age 42 yo ± 17) undergoing elective plastic surgery was collected. Tissue was digested for 30 min at 37 °C with 0.075% w/v type I collagenase (Worthington Biochemical Co., Lakewood, NJ, USA) and filtered with a 100 µm cell strainer. Cells were recovered by centrifugation (1000× *g*, 5 min) and seeded at 10 × 10^3^ cells/cm^2^ in DMEM/F12 (ThermoFisher, Waltham, MA, USA) supplemented with 10% FBS and 1% PSG (ThermoFisher). ASCs able to adhere to plastic were cultivated at 37 °C, 5% CO_2_, and 95% humidity.

Amniotic membrane fragments (3 × 3 cm^2^) underwent digestion with dispase at 37 °C and were subsequently transferred to RPMI 1640 complete medium (Sigma-Aldrich, St. Louis, MO, USA) to halt the digestion process. Following this, the fragments were incubated in collagenase, and the resulting supernatant, obtained after centrifugation, was filtered using a 100 μm cell strainer (BD Falcon, Bedford, MA, USA), with cells then harvested through centrifugation. The freshly isolated cells (p0) were seeded at a density of 10^4^ cells/cm^2^ and expanded until passage 1 (p1) in Chang medium C (Irvine Scientific, Santa Ana, CA, USA), supplemented with 2 mM L-glutamine, and maintained at 37 °C in a 5% CO_2_ incubator. The hAMSCs utilized in this study underwent phenotypic characterization after isolation (p0) [45], meeting the essential criteria for mesenchymal stromal cells (MSCs) [46,47,48].

### 4.3. ASC, BMSC, and hAMSC Immunophenotype by Flow Cytometry

ASCs, BMSCs, and hAMSCs at passage 1 were analyzed by flow cytometry to score the expression of hemato-endothelial (CD45-PE Vio770 clone REA747; Miltenyi Biotec, Bergisch Gladbach, Germany. CD31-APC clone WM59; Biolegend, San Diego, CA, USA) and MSC (CD73-PE clone REA804 and CD90-FITC clone REA897; Miltenyi Biotec, Bergisch Gladbach, Germany) markers. A minimum of 30,000 events were acquired with a CytoFLEX flow cytometer (Beckman Coulter, Fullerton, CA, USA).

### 4.4. RNA Extraction and mRNA Profiling

An miRNeasy Micro Kit (Qiagen, Hilden, Germany), following the manufacturer’s protocol, was used to isolate total RNA from cells at passage 1. Isolated RNA was quantified, and equal amounts for each sample were reverse transcribed with an iScript™ cDNA Synthesis Kit (BioRad, Hercules, CA, USA). After cDNA synthesis, the reaction was preamplified with SsoAdvanced™ PreAmp Supermix (BioRad). qRT-PCR reactions were performed with iTaq Universal SYBR Green Supermix (BioRad) in a CFX Opus Real-Time PCR Systems (BioRad), following the manufacturer’s protocol. Five HKGs were tested: *ACTB* (Actin Beta), *EF1A* (Eukaryotic Translation Elongation Factor 1 Alpha 1), *RPLP0* (Ribosomal Protein Lateral Stalk Subunit P0), *TBP* (TATA-Box Binding Protein), and *GAPDH* (Glyceraldehyde-3-Phosphate Dehydrogenase). Two OA-related genes were tested: *ICAM1* (Intercellular Adhesion Molecule 1) and *IL8* (Interleukin-8). Primer sequences will be provided upon request.

### 4.5. Data Analysis

Four applets were selected to score HKG stability: NormFinder [49], BestKeeper [50], geNorm [51], and the comparative ΔCt method [52]. Normfinder allows the definition of a stability value (low value for high stability) by relying on linear-scale quantitative data. BestKeeper uses the standard deviation (SD) (low value for high stability). geNorm provides an M-value based on the average pairwise expression ratio (stability defined by M < 1.5). Eventually, “pairs of genes” are compared in the ΔCt approach. A HKG stability ranking is generated by each approach, with a series of continuous integers starting from 1. The four rankings were computed by RefFinder, a web-based comprehensive tool that assigns an appropriate weight to an individual HKG, calculating the geometric mean of the different rankings to generate the overall final ranking [53].

### 4.6. Hierarchical Clustering and Principal Component Analysis

Principal component analysis (PCA) and hierarchical clustering of the Ct values were obtained with the ClustVis webtool [54]. Settings for data analyses were (i) data pre-processing with no transformation, centering, and no scaling; (ii) and a heat map with the correlation as the clustering distance for rows and columns, with the average as the clustering method for rows and columns, with a tighter cluster first for tree ordering for rows and columns.

### 4.7. Statistical Analyses

GraphPad Prism Software version 8.0.2 (GraphPad, San Diego, CA, USA) was used for statistical analyses. Normal data distribution was tested with a Shapiro–Wilk normality test (α of 0.01). For values passing the normality test, for two datasets, an unpaired *t*-test was performed, while for three datasets, a repeated measures one-way ANOVA was performed with Tukey’s post hoc test. The level of significance was set at *p*-value ≤ 0.05.

## 5. Conclusions

In this work, a dichotomy in the reliability of HKGs for adult and fetal/neonatal sources emerged, with a complete inversion of stability order. HKG choice was more impactful when comparing single donors, while it appeared less stringent when sources were analyzed side-by-side. These results contribute valuable information to guide the selection of the most appropriate MSC type or orthobiologics for musculoskeletal diseases, especially in the context of OA. The described outcomes are also intended to be a stimulus for basic researchers and clinicians to analyze cells and treatments under the light of the most reliable gene expression approach.

## Figures and Tables

**Table 1:** Mean Ct values of HKGs analyzed in this study (mean ± SEM, n = 12 for “MSCs” and n = 4 for “BMSCs, ASCs, and hAMSCs”).

	*ACTB*	*EF1A*	*GAPDH*	*RPLP0*	*TBP*
MSCs	11.90 ± 0.14	10.57 ± 0.12	11.37 ± 0.13	11.55 ± 0.14	20.55 ± 0.10
BMSCs	12.06 ± 0.17	10.63 ± 0.07	11.57 ± 0.07	11.87 ± 0.10	20.64 ± 0.23
ASCs	12.10 ± 0.25	10.24 ± 0.16	11.41 ± 0.33	11.09 ± 0.16	20.67 ± 0.07
hAMSCs	11.53 ± 0.16	10.84 ± 0.24	11.12 ± 0.08	11.68 ± 0.26	20.35 ± 0.11

**Table 2:** Stability rankings of the tested HKGs.

Source	Geomean		Delta CT		BestKeeper		NormFinder		Genorm	
**BMSCs**	*EF1A*	1.19	*RPLP0*	0.30	*EF1A*	0.12	*EF1A*	0.08	*EF1A*|*RPLP0*	0.17
	*RPLP0*	1.57	*EF1A*	0.31	*GAPDH*	0.12	*RPLP0*	0.08		
	*ACTB*	3.22	*ACTB*	0.40	*RPLP0*	0.16	*ACTB*	0.29	*ACTB*	0.22
	*GAPDH*	3.36	*GAPDH*	0.44	*ACTB*	0.29	*GAPDH*	0.36	*GAPDH*	0.29
	*TBP*	5.00	*TBP*	0.58	*TBP*	0.39	*TBP*	0.55	*TBP*	0.41
**ASCs**	*EF1A*	1.32	*EF1A*	0.33	*TBP*	0.12	*EF1A*	0.08	*EF1A*|*RPLP0*	0.17
	*TBP*	1.86	*TBP*	0.40	*RPLP0*	0.25	*TBP*	0.2		
	*RPLP0*	2.06	*RPLP0*	0.41	*EF1A*	0.26	*RPLP0*	0.28	*TBP*	0.23
	*ACTB*	4.00	*ACTB*	0.48	*ACTB*	0.49	*ACTB*	0.36	*ACTB*	0.32
	*GAPDH*	5.00	*GAPDH*	0.66	*GAPDH*	0.55	*GAPDH*	0.62	*GAPDH*	0.46
**hAMSCs**	*GAPDH*	1.32	*GAPDH*	0.50	*GAPDH*	0.16	*GAPDH*	0.19	*ACTB|TBP*	0.23
	*TBP*	1.68	*TBP*	0.54	*TBP*	0.18	*TBP*	0.34		
	*ACTB*	2.45	*ACTB*	0.63	*ACTB*	0.26	*EF1A*	0.54	*GAPDH*	0.34
	*EF1A*	3.72	*EF1A*	0.67	*EF1A*	0.40	*ACTB*	0.55	*EF1A*	0.55
	*RPLP0*	5.00	*RPLP0*	0.70	*RPLP0*	0.43	*RPLP0*	0.59	*RPLP0*	0.61
**BMSCs/ASCs**	*EF1A*	1.00	*EF1A*	0.38	*EF1A*	0.23	*EF1A*	0.13	*EF1A|RPLP0*	0.26
	*RPLP0*	2.59	*ACTB*	0.50	*TBP*	0.25	*ACTB*	0.35		
	*ACTB*	2.63	*RPLP0*	0.50	*GAPDH*	0.37	*RPLP0*	0.38	*ACTB*	0.39
	*TBP*	3.36	*TBP*	0.54	*ACTB*	0.39	*TBP*	0.42	*TBP*	0.45
	*GAPDH*	4.40	*GAPDH*	0.56	*RPLP0*	0.39	*GAPDH*	0.46	*GAPDH*	0.49
**BMSCs/hAMSCs**	*GAPDH*	1.00	*GAPDH*	0.49	*GAPDH*	0.22	*GAPDH*	0.26	*ACTB|GAPDH*	0.41
	*TBP*	2.71	*RPLP0*	0.53	*EF1A*	0.28	*RPLP0*	0.36		
	*RPLP0*	2.83	*TBP*	0.57	*TBP*	0.28	*TBP*	0.41	*TBP*	0.47
	*ACTB*	2.99	*ACTB*	0.57	*RPLP0*	0.32	*ACTB*	0.45	*RPLP0*	0.53
	*EF1A*	4.40	*EF1A*	0.58	*ACTB*	0.39	*EF1A*	0.46	*EF1A*	0.55
**ASCs/hAMSCs**	*TBP*	1.00	*TBP*	0.57	*TBP*	0.18	*TBP*	0.30	*ACTB|TBP*	0.38
	*GAPDH*	2.21	*GAPDH*	0.64	*GAPDH*	0.32	*GAPDH*	0.42		
	*ACTB*	2.99	*EF1A*	0.66	*RPLP0*	0.38	*EF1A*	0.51	*GAPDH*	0.47
	*EF1A*	3.46	*ACTB*	0.69	*EF1A*	0.42	*ACTB*	0.56	*EF1A*	0.62
	*RPLP0*	4.40	*RPLP0*	0.69	*ACTB*	0.43	*RPLP0*	0.57	*RPLP0*	0.65
**ALL**	*TBP*	1.41	*TBP*	0.57	*TBP*	0.25	*TBP*	0.38	*EF1A|RPLP0*	0.32
	*EF1A*	2.06	*EF1A*	0.57	*GAPDH*	0.33	*GAPDH*	0.38		
	*GAPDH*	2.45	*GAPDH*	0.58	*EF1A*	0.34	*EF1A*	0.41	*GAPDH*	0.53
	*RPLP0*	2.83	*RPLP0*	0.60	*RPLP0*	0.40	*RPLP0*	0.47	*TBP*	0.57
	*ACTB*	5.00	*ACTB*	0.61	*ACTB*	0.42	*ACTB*	0.47	*ACTB*	0.59

All stands for all conditions analyzed together.

## Data Availability

Raw data for this study are available at https://osf.io/5aqcw/?view_only=cb5fc5c4bfe441e7a8544043c109be2d (generated on 23 November 2023).
